# Comparison of antipsychotic drug use among Dutch Youth before and after implementation of the Youth Act (2010–2019)

**DOI:** 10.1007/s00787-022-01949-0

**Published:** 2022-02-09

**Authors:** Y. Bais, R. A. Hermans, C. C. M. Schuiling-Veninga, H. J. Bos, S. M. Kloosterboer, B. C. M. de Winter, M. Simoons, G. C. Dieleman, M. H. J. Hillegers, B. C. P. Koch, B. Dierckx

**Affiliations:** 1 Department of Hospital Pharmacy, Erasmus Medical Center, Rotterdam, The Netherlands; 2 Department of Child and Adolescent Psychiatry/Psychology, Erasmus Medical Center, Rotterdam, The Netherlands; 3 Department of Pharmacotherapy, Epidemiology and Economics, University of Groningen, Groningen, The Netherlands

**Keywords:** Youth act, Antipsychotics, Prevalence, Incidence, Dosages, Duration of use

## Abstract

**Objective:**

The Dutch law on youth care (the Youth Act) was implemented from 2015 onwards. One of the government’s aims by implementing this new policy was de-medicalization of youths by separating youth mental healthcare from the rest of the healthcare system. A previous study conducted by our research group showed that prevalence rates of antipsychotic drug prescriptions stabilized among Dutch youth in the period 2005–2015, just before the introduction of the Youth Act. In our study, we aimed to describe antipsychotic drug use among Dutch children aged 0–19 years old before and after implementation of the Youth Act (2010–2019).

**Methods:**

We analyzed prescription data of 7405 youths aged 0–19 years using antipsychotic drugs between 2010 and 2019, derived from a large Dutch community pharmacy-based prescription database (IADB.nl).

**Results:**

Prevalence rates of antipsychotic drug use per thousand youths decreased significantly in youths aged 7–12 years old in 2019 compared to 2015 (7.9 vs 9.0 *p* < 0.05). By contrast, prevalence rates increased in adolescent females in 2019 compared to 2015 (11.8 vs 9.5 *p* < 0.05). Incidence rates increased significantly in adolescent youths in 2019 compared to 2015 (3.9 vs 3.0 *p* < 0.05), specifically among adolescent girls (4.2 per thousand in 2019 compared to 3.0 per thousand in 2015). Dosages in milligram declined for the most commonly prescribed antipsychotic drugs during the study period. The mean duration of antipsychotic drug use in the study period was 5.7 (95% CI 5.2–6.2) months.

**Conclusion:**

Despite the aim of the Youth Act to achieve de-medicalization of youths, no clear reduction was observed in prevalence rates of antipsychotic drugs or treatment duration in all subgroups. Prevalence rates even increased in adolescent females.

**Supplementary Information:**

The online version contains supplementary material available at 10.1007/s00787-022-01949-0.

## Introduction

Child and adolescent psychiatry and youth mental health services have evolved in many ways in the past few decades across European countries [1]. Policy changes regarding mental health care for children and youth have also taken place in the Netherlands [2]. In 2015, the Dutch government implemented reform of care for children and families, with major consequences for youth mental health care. The law on youth care (the Youth Act) indicates that municipalities now have the responsibility for funding and organization of all kinds of youth care. An important aim of the reform is to stimulate people to ‘normalize’ behavioral problems in children and to make less use of expensive specialized treatment. Instead, more use should be made of low-key preventive care with a focus on strengthening the pedagogical climate of youths [3].

Most municipalities (87%) in the Netherlands have implemented this policy by creating multidisciplinary youth care teams that provide community-based care to children and their families [4]. The municipalities are responsible for determining the composition of these youth care teams, which may consist of, for example, social workers and behavioral coaches. These Youth care teams should identify problems and assist children and their parents in solving their problems with the help of the patients’ social network. A similar approach in some Canadian provinces resulted in high patient satisfaction and improved access to health care services, although long waiting times for mental health services and limited programming for children and youth with mental health problems were not resolved [5].

One of the central aims of the Youth Act is ‘de-medicalization’ [6]. De-medicalization should be interpreted as an overall reduction in the use of psychiatric facilities, including reduced prescriptions of psychotropic medication. It is, however, questionable whether this reorganization of care indeed has led to less psychotropic drug use. In the first evaluation of the Youth Act, uncertainties were raised about the Youth care teams regarding the composition of the teams and their working method [4]. In particular, it is not documented which requirements are set for the expertise of the members of the teams, possibly leading to suboptimal triage of children with serious mental illnesses. However, a thorough evaluation of the effects of the Youth Act on prescribing trends in youth mental health care is currently lacking.

Trends in antipsychotic drug use among youths following the Youth Act are of particular interest. A previous study conducted by our research group investigated antipsychotic drug use among Dutch youth in the period 2005 to 2015, just before implementation of the Youth Act [7]. We found that prevalence rates of antipsychotic drug prescriptions were high but stabilized and that antipsychotic drugs are most commonly prescribed by specialists. Prescribing trends of antipsychotic drugs in the period after the implementation of the Youth Act in 2015 are lacking, but highly needed to assess whether a community-based mental health care system for youths may indeed lead to ‘de-medicalization’ in terms of reduced prescriptions of psychotropic medication.

Therefore, we aim to describe prescription patterns and trends of antipsychotic use among youths in different age groups in the Netherlands before (between 2010 and 2015) and after (between 2015 and 2019) the implementation of the Youth Act.

## Methods

### Data source

Data from the population-based prescription database IADB.nl served as the basis for this study. The IADB database comprises prescription drug dispending data from approximately 100 community pharmacies in the northern and eastern part of the Netherlands from 1994 onwards and has been proven to be representative on a national level [8]. The database covers an estimated population of 900,000 patients. Registration in the database is irrespective of health care insurance and prescriber. In-hospital prescriptions are not included in the database. The total population estimates were extracted from general population statistics from the Dutch Central Bureau for Statistics (CBS).

The study database IADB.nl comprises de-identified records and data are collected in accordance with the national and European guidelines on privacy requirements for handling human data. Therefore, approval of the medical ethics committee was not required.

### Study sample

We selected youths aged 0–19 years old who were prescribed an antipsychotic drug between January 1, 2010 and December 31, 2019. We used the World Health Organization’s Anatomical Therapeutic Chemical/Defined Daily Dose Classification System to define antipsychotic drugs as class N05A, N05AN (lithium) excluded.

### Data analysis

#### Prevalence and incidence

Prevalence and incidence rates were calculated per year over a period of 10 years from 2010 to 2019, stratified by gender and age groups (0–6 years, 7–12 years, 13–19 years). In the database, the age on the first of January of the year of the prescription was used. A youth was considered to be a new user when they had been present in the database for at least 90 days before the first antipsychotic drug prescription. Prevalence and incidence rates were calculated by dividing the number of users over the estimated total population and are expressed per thousand (CBS data).

#### Dose analysis

Dose analysis was performed using the four most commonly prescribed antipsychotic drugs. Defined daily doses [9] were used to analyze mean dosages per year. Prescriptions issued for less than 7 days were excluded from analysis to exclude rescue medication. Pipamperone was excluded from dose analysis as this is often prescribed as a 40 mg/ml liquid formulation and daily dose was not consequently processed in the database as milliliters (ml) or milligrams (mg). Means are presented as value ± the standard deviation (SD).

#### Duration of use

Duration of antipsychotic drug use in months was calculated by median survival times using a Kaplan Meier estimator. The start of an episode of antipsychotic drug use was defined as described above. The episode was considered to have ended if the number of days for which medication was prescribed plus 90 days had passed and the user could still be followed in the database. All cases for which the start and/or end of an episode could not be determined were censored. Youths who were new users in 2019 were excluded from analysis because of high rates of censoring. Duration of use was stratified by gender, age groups and start year. Subgroups were compared using the Log-rank test.

### Statistical analysis

Differences were considered significant at *p* < 0.05. Statistical analyses were performed with SPSS for Windows, version 25 and Microsoft Excel 2016. The 95% confidence intervals were calculated using the Score Method with continuity correction [10]. Proportions were compared using the Chi square test.

## Results

The total population aged 0–19 years ranged from 175,672 persons in 2010 to 185,006 persons in 2019. Between 2010 and 2019, a total of 7405 youths aged 0–19 years present in the IADB database were prescribed antipsychotic drugs.

### Prevalence

The 1-year prevalence rates of antipsychotic drug prescriptions were 8.9 (95% CI 8.5–9.4) in 2010 and 8.5 (95% CI 8.1–8.9) per thousand youths in 2019. The prevalence rates stratified by age, gender and year are presented in Table 1. In 2017, 2 years after implementation of the Youth Act, the overall prevalence was lowest at 7.9 per thousand youths. Prevalence rates post-implementation significantly decreased in the total group of 7–12 year old children, from 9.0 (95% CI 8.3–9.8) per thousand in 2015 to 7.9 (95% CI 7.2–8.7) per thousand in 2019. By contrast, significantly higher prevalence rates were observed in girls between 13 and 19 years old. Boys were more likely to use antipsychotic drugs compared to girls in all years irrespective of age group.

**Table 1:** Prevalence (per thousand) of antipsychotic drug prescriptions among Dutch youth up to 19 years

	2010^a^		2015^b^		2019^c^	
	per 1000	95% CI	per 1000	95% CI	per 1000	95% CI
*Total*						
All ages	8.9	(8.5–9.4)	8.4	(8.0–8.8)	8.5	(8.1–8.9)
0–6	1.4	(1.1–1.7)	0.9*	(0.6–1.1)	0.8	(0.5–1.0)
7–12	12.2	(11.2–13.1)	9.0*	(8.3–9.8)	7.9°	(7.2–8.7)
13–19	13.1	(12.3–14.0)	14.1	(13.3–15.0)	14.7	(13.8–15.6)
*Males*						
0–6	2.2	(1.7–2.8)	1.1*	(0.8–1.5)	1.1	(0.7–1.5)
7–12	18.8	(17.1–20.4)	13.2*	(11.9–14.5)	11.7	(10.4–12.9)
13–19	19.0	(17.5–20.5)	18.9	(17.5–20.2)	17.5	(16.2–18.9)
*Females*						
0–6	0.6	(0.3–0.8)	0.6	(0.3–0.9)	0.4	(0.2–0.7)
7–12	5.3	(4.4–6.2)	4.6	(3.8–5.4)	4.0	(3.2–4.7)
13–19	7.6	(6.6–8.5)	9.5*	(8.6–10.5)	11.8°	(10.8–12.9)

^a^*n* = 88,912 boys, *n* = 86,760 girls

^b^*n* = 102,820 boys, *n* = 99,231 girls

^c^*n* = 93,694 boys, *n* = 91,312 girls

*p < 0.05, significantly different compared to 2010

°*p* < 0.05, significantly different compared to 2015

**Fig. 1:**
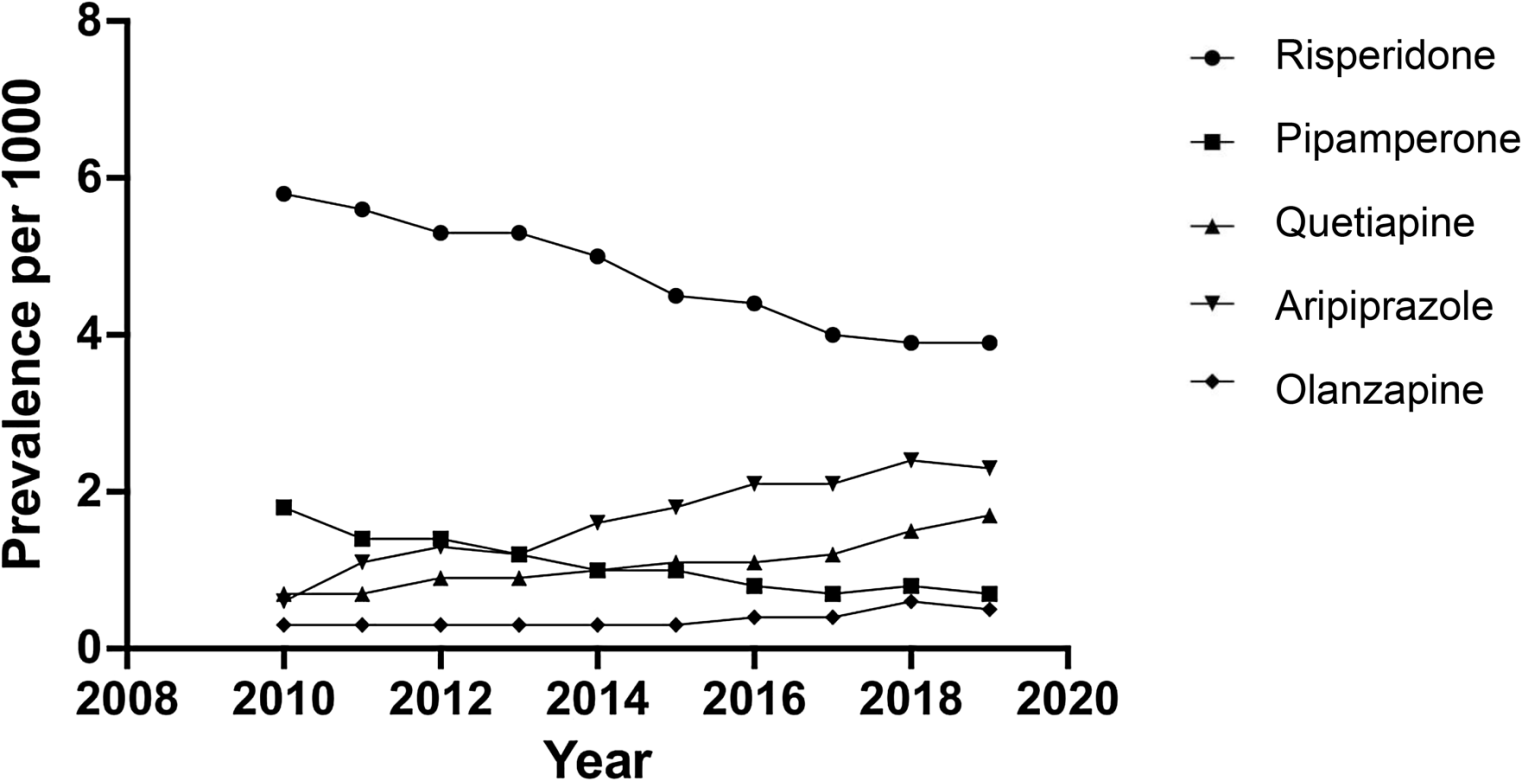
Prevalence of antipsychotic drug prescriptions in youth aged 0–19 years

The most commonly prescribed antipsychotic drugs were risperidone, aripiprazole, quetiapine, pipamperone and olanzapine (Fig. 1). In total, these antipsychotic drugs accounted for almost 95% of all prescribed antipsychotic drugs. Risperidone was the most frequently prescribed antipsychotic drug in all years with prevalence rates per 1000 youths ranging from 5.8 (95% CI 5.5–6.2) in 2010 to 3.9 (95% CI 3.6–4.1) in 2019. This downward trend was also observed for pipamperone, ending in a prevalence rate of 0.7 per 1000 in 2019. Both quetiapine and aripiprazole showed an upward trend that continued after introduction of the Youth Act. Prevalence rates of the most commonly used antipsychotic drugs in 2015 and 2019 were stratified by age and gender (see Supplementary Tables 1a–c). Notably, in 2019, quetiapine and olanzapine were by far most commonly prescribed to adolescent girls.

### Incidence

The overall incidence rate was 2.3 per thousand youths (95% CI 2.1–2.6) in 2019 compared to 2.0 (95% CI 1.8–2.2) in 2015 and 2.4 (95% CI 2.2–2.7) in 2010. Incidence was lowest in 2016 at 1.8 (95% CI 1.6–2.0). The incidence rates, stratified by age, gender and start year are presented in Table 2. Overall, new users aged 13–19 increased significantly in 2019 compared to 2015. Significantly more girls between 13 and 19 years old started antipsychotic drug use in 2019 compared to 2015.

**Table 2:** Incidence per thousand of antipsychotic drug prescriptions among children up to 19 years

	2010^a^		2015^b^		2019^c^	
	per 1000	95% CI	per 1000	95% CI	per 1000	95% CI
Total						
All ages	2.4	(2.2–2.7)	2.0	(1.8–2.2)	2.3	(2.1–2.6)
0–6	0.8	(0.6–1.1)	0.4*	(0.3–0.6)	0.4	(0.3–0.6)
7–12	3.5	(3.0–4.0)	2.2*	(1.9–2.6)	2.1	(1.7–2.5)
13–19	3.1	(2.6–3.5)	3.0	(2.6–3.4)	3.9°	(3.5–4.4)
Males						
0–6	1.3	(0.9–1.7)	0.6*	(0.3–0.8)	0.7	(0.4–1.0)
7–12	5.2	(4.3–6.0)	2.9*	(2.3–3.6)	2.9	(2.3–3.6)
13–19	3.9	(3.3–4.6)	3.0*	(2.4–3.5)	3.6	(2.4–3.5)
Females						
0–6	0.3	(0.1–0.5)	0.3	(0.1–0.5)	0.2	(0.0–0.3)
7–12	1.7	(1.2–2.3)	1.5	(1.0–2.0)	1.3	(0.8–1.7)
13–19	2.2	(1.7–2.7)	3.0*	(2.5–3.6)	4.2°	(3.6–4.9)

^a^*n* = 88,912 boys, *n* = 86,760 girls

^b^*n* = 102,820 boys, *n* = 99,231 girls

^c^*n* = 93,694 boys, *n* = 91,312 girls

*p < 0.05, significantly different compared to 2010

°*p* < 0.05, significantly different compared to 2015

### Dose analysis

There was a total of 16,541 prescriptions for the four most commonly prescribed antipsychotic drugs, on which analysis of the dosages (in milligram) could be performed.

The mean daily dosage (± standard deviation) for risperidone was 1.1 mg (± 0.96) in 2010 compared to 0.9 mg (± 0.86) in 2019. The mean daily dosage of aripiprazole decreased from 10.1 mg (± 5.7) in 2010 to 3.5 mg (± 3.0) in 2019. For quetiapine, the mean daily dosage also decreased from 91.7 mg (± 102.8) in 2010 to 42.8 mg (± 48.1) in 2019. Mean daily olanzapine dosages fluctuated and were highest in 2011 (7.7 mg ± 5.2) and lowest in 2017 (4.9 mg ± 3.7). Mean daily dosages in 2010, 2015 and 2019 were stratified by age and gender (see Supplementary Table 2). Notably, mean daily quetiapine dosages decreased from 91.6 in 2010 to 42.6 in 2019 among adolescents.

### Duration of use

**Fig. 2:**
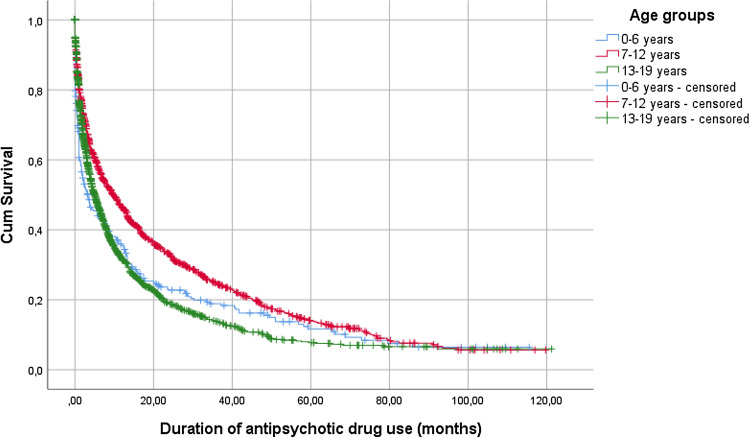
Duration of antipsychotic drug use in youths aged 0–19 years

The overall median duration of antipsychotic drug use was 5.7 months (95% CI 5.2–6.2). Results for various subgroups are presented in Table 3. Median duration of therapy increased for antipsychotic drug treatment initiated in 2010 compared to 2018, from 4.0 months (95% CI 2.8–5.2) to 7.2 months (95% CI 5.1–9.2). Therapy duration was longest for children aged 7–12 years in all start years. Antipsychotics were consistently used longer by boys compared to girls, although the duration of use in girls seems to be increasing. Survival rates are visualized in Fig. 2.

**Table 3:** Duration of use (in months) of antipsychotic drugs among children up to age 19 years

	Median	95% CI
All users*	5.7	(5.2–6.2)
*Age (years)*		
0–6	3.2	(1.3–5.2)
7–12	9.1	(7.6–10.7)
13–19	4.4	(3.9–4.9)
*Gender*		
Boys	6.9	(6.0–7.7)
Girls	4.2	(3.6–4.7)
*Start year*		
2010	4.0	(2.8–5.2)
2015	5.8	(4.4–7.2)
2018	7.2	(5.1–9.2)
Agent**		
Risperidone	12.6	(9.5–15.7)
Aripiprazole	9.3	(5.4–13.2)
Quetiapine	3.4	(2.6–4.2)

*Analysis was performed for the years 2010–2018

**Analysis was performed for the years 2016–2018

## Discussion

According to our findings, the Dutch reform of the mental health care system for children and youths in 2015 has not led to fewer antipsychotic prescriptions or to shorter treatment duration. From 2010 to 2019, prevalence rates of antipsychotic drug use in the Netherlands fluctuated around 9 users per thousand youths with the lowest number of users in 2017 (7.9 per thousand youths). We observed a decrease in mean dosage for the most commonly prescribed antipsychotics. Furthermore, the median duration of antipsychotic drug use increased with longest duration of use for treatments initiated in 2018. Our findings are in accordance with the first interim evaluation of the Youth Act, which reported no reduction in the use of specialized care services [4].

The stabilization of antipsychotic drug use in youth overall, that had been described in the Netherlands from 2005 to 2015 [7], continued after implementation of the Youth Act in 2015 until 2019. However, prevalence rates decreased in children between 7 and 12 years old. In contrast to patients in middle childhood, prevalence rates increased in adolescent girls. This finding is consistent with a recent study that reported increased antipsychotic use in this subgroup in Finland [11]. The contrasting prevalence changes in primary school aged children compared to adolescent youths might be explained by differences in distribution of diagnoses among different age groups [12, 13]. Patients in middle childhood, mainly diagnosed with behavior disorders, may benefit from social interventions initiated by youth care teams resulting in decreased antipsychotic use in this subgroup. By contrast, adolescent patients mainly face more severe mental health problems that require specialized treatments for which there are often long waiting lists, which might lead to a search for a quick and effective solution [14]. In this group, where entry into specialized care may be delayed by pedagogical or social interventions, situations may be created in which antipsychotic drug treatment is deemed necessary as part of a crisis intervention, resulting in higher prevalence rates post-implementation of the Youth Act.

Further exploration of our data on the increase in prevalence rates among adolescent girls shows that increased prescription rates for quetiapine in this group seem to be a major contributing factor. Quetiapine belongs to the second-generation antipsychotics and is frequently used in child and adolescent psychiatric practice [15]. We found that in 2019, 61.5% of the quetiapine users in our sample were adolescent girls. Meanwhile, the mean dosage of quetiapine decreased in this subgroup, from 87.6 mg in 2010 to 38.2 mg in 2019 (Supplementary Table 2). These findings most likely reflect off-label use. For example, short-duration treatment of quetiapine in low dosages (i.e., 25–75 mg) is often used to treat insomnia in girls with diagnoses of anxiety disorder, eating disorder, borderline personality disorder, and depressive disorders [16]. A study on prescribing of antipsychotics in primary care in the UK found that low doses of quetiapine are also often prescribed to females diagnosed with anxiety or depression [17] and a recent Danish study reported increased use of low-dose quetiapine outside its approved indications [18]. It is possible that over the years, an increasing number of adolescent girls face unmet medical needs ending in crisis situations where quetiapine use is required. This is supported by the finding that treatment duration among adolescent girls has decreased.

Despite a higher prescription rate of quetiapine post-implementation compared to the period before the Youth Act came into effect, risperidone remained the most commonly prescribed antipsychotic drug in all years. This is comparable to other European countries [19–21]. However, risperidone prescriptions decreased from 2010 until 2019, which was also previously described from 2005 till 2015 [7]. Meanwhile, aripiprazole prescriptions increased over time resulting in aripiprazole being the second-most commonly prescribed antipsychotic drug in 2019. A possible explanation for this trend might be that clinicians expect that aripiprazole, because of its unique mechanism of action, causes less weight gain compared to other second-generation antipsychotics, although studies have shown contrasting results [22, 23]. Lastly, higher prevalence rates of aripiprazole may also be explained by increased familiarity among specialists with prescribing aripiprazole to children and adolescents since its introduction on the market in 2002.

We observed an increase in median treatment duration over the years. Boys showed a significantly longer treatment duration than girls, which was consistent with earlier findings [20, 24, 25], although duration of use in girls seemed to be increasing in our study period when compared to the previous study by Kloosterboer et al., in which analysis was performed for the years 2005–2014 [7].

In our study, we observed a trend toward lower dosages of antipsychotic drugs from 2010 to 2019, with the exception of olanzapine. This finding has been observed before in a Scandinavian study conducted in children, adults and the elderly [24]. A possible explanation might be increased use of most antipsychotic drugs outside main indications, while olanzapine might mostly be prescribed to treat severe mental illness. In addition, in the past decade, more studies have been conducted that focus on serious side effects related to antipsychotic drug use [26, 27], which may have been an incentive to prescribe lower dosages. This development prompts further investigation of the indications for initiating antipsychotic drugs.

This study was the first study to report on antipsychotic drug use after the implementation of the Youth Act, reflecting broader developments worldwide to organize youth mental health services in a community-based manner. We used a large dataset in a country that is representative for the Western world. Moreover, we were able to conduct analyses on dosages and duration of drug use with no limitations regarding to type of health insurance or healthcare provider.

The results of this study must be considered in the context of its limitations. First, no information about indications for medication use was available, which could be a valuable addition to prescription data to give explanations for the observed changes. Second, the IADB database only includes parts of the Netherlands, representing mainly less urbanized areas. Some literature suggests antipsychotic drug prescriptions might decrease with greater medical density [28]. Hypothesizing that urban regions contain more medical facilities, this might have led to an overestimation of the prescription rates found in our study. However, the IADB database has previously proven to be representative for the whole country [8]. Third, prescription rates do not represent actual usage rates. Moreover, we had no information about treatment adherence. Fourth, by our definition of a new user, episodic use by the same user could also be defined as a new user, which might have led to an overestimation of incidence rates. Also, as our analysis does not cover in-hospital use, children that were hospitalized for more than 90 days might have been defined as a new user after discharge from the hospital. This fact could have led to an overestimation of incidence rates and an underestimation of duration of use. However, hospitalization in child and adolescent psychiatry is not very common [29].

While the implementation of the Dutch Youth Act did not change the overall prevalence rate of antipsychotic drug use, there were changes in prevalence rates in different age and gender groups. While these changes may be due to the changes in youth care, research linking prescriptions to indications is needed to draw firm conclusions. Furthermore, for a more complete evaluation of the impact of the Youth Act, the use of other medical resources, such as psychiatric hospital admissions and emergency room visits, should also be investigated. Suggestions for future research also include further investigation of different policy strategies of different municipalities and different compositions of youth care teams in relation to drug prescriptions, referrals to and waiting times for specialized care, and patient satisfaction. Since the implementation of the Youth Act was accompanied by financial cutbacks in psychiatric youth care, it is also important to consider the impact of these cutbacks on psychiatric care.

## Conclusion

The reorganization of youth mental health care in a community-based setting with the Dutch Youth Act did not lead to fewer antipsychotic drug prescriptions or shorter durations of use in children and adolescents in the Netherlands between 2010 and 2019. The use of low-dose quetiapine increased, especially in adolescent girls, which suggests increasing off-label use among this subgroup.

## Supplementary Information

Below is the link to the electronic supplementary material.

Supplementary file1 (DOCX 42 KB)

## Data Availability

The data that support the findings of this study are available in the database IADB.nl. However, these data are only made available after approval of a study protocol.

## Code availability

Syntaxes used to analyze the data can be made available upon reasonable request.
