# Supplementary material for: Comparison of antipsychotic drug use among Dutch Youth before and after implementation of the Youth Act (2010–2019)
Source: Eur Child Adolesc Psychiatry. 2022 Feb 9;32(8):1427–34. doi: 10.1007/s00787-022-01949-0 (PMC10326153; doi:10.1007/s00787-022-01949-0)
Supplement: Supplementary file 1 — Supplementary file1 (DOCX 42 KB) [file 787_2022_1949_MOESM1_ESM.docx]

**Supplementary tables and figures**

Table 1a. Type of users antipsychotic drugs in 2010, 2015 and 2019

| **Risperidone** | | | |
| --- | --- | --- | --- |
|  | **2010** | **2015** | **2019** |
| Males 0-6 | 4.6% | 1.6% | 2.1% |
| Males 7-12 | 37.4% | 29.5% | 28.2% |
| Males 13-19 | 37.1% | 44.9% | 42.6% |
|  |  |  |  |
| Females 0-6 | 0.9% | 1.0% | 0.6% |
| Females 7-12 | 7.9% | 8.5% | 8.8% |
| Females 13-19 | 12.1% | 14.4% | 17.7% |
|  |  |  |  |
| **Aripiprazole** | | | |
|  | **2010** | **2015** | **2019** |
| Males 0-6 | 0.9% | 1.9% | 0.9% |
| Males 7-12 | 25.7% | 25.2% | 22.6% |
| Males 13-19 | 44.0% | 36.7% | 45.3% |
|  |  |  |  |
| Females 0-6 | 0.0% | 0.3% | 0.0% |
| Females 7-12 | 9.2% | 9.4% | 5.3% |
| Females 13-19 | 20.2% | 26.5% | 25.9% |
|  |  |  |  |
| **Pipamperone** | | | |
|  | **2010** | **2015** | **2019** |
| Males 0-6 | 7.0% | 5.1% | 6.5% |
| Males 7-12 | 32.3% | 24.6% | 23.6% |
| Males 13-19 | 33.5% | 42.1% | 37.4% |
|  |  |  |  |
| Females 0-6 | 1.3% | 2.1% | 1.6% |
| Females 7-12 | 11.5% | 11.8% | 13.0% |
| Females 13-19 | 14.4% | 14.4% | 17.9% |
|  |  |  |  |
| **Quetiapine** | | | |
|  | **2010** | **2015** | **2019** |
| Males 0-6 | 0.0% | 0.5% | 0.3% |
| Males 7-12 | 6.5% | 0.9% | 3.1% |
| Males 13-19 | 44.4% | 42.3% | 32.9% |
|  |  |  |  |
| Females 0-6 | 0.8% | 0.5% | 0.3% |
| Females 7-12 | 4.0% | 0.0% | 1.9% |
| Females 13-19 | 44.4% | 55.8% | 61.5% |
|  |  |  |  |
| **Olanzapine** | | | |
|  | **2010** | **2015** | **2019** |
| Males 0-6 | 3.6% | 4.8% | 0.0% |
| Males 7-12 | 7.3% | 0.0% | 7.9% |
| Males 13-19 | 43.6% | 50.0% | 38.6% |
|  |  |  |  |
| Females 0-6 | 0.0% | 0.0% | 0.0% |
| Females 7-12 | 10.9% | 1.6% | 2.0% |
| Females 13-19 | 34.5% | 43.5% | 51.5% |

Table 2. Mean dosages in mg of most commonly used antipsychotic drugs stratified by age and gender in 2010, 2015 and 2019.

|  | **2010** | **2015** | **2019** |
| --- | --- | --- | --- |
| **Risperidone** |  |  |  |
| 0-6 | 0.84 ± 1.00 | 0.67 ± 0.39 | 0.89 ± 1.25 |
| 7-12 | 0.84 ± 0.83 | 0.91 ± 1.37 | 0.73 ± 0.80 |
| 13-19 | 1.27 ± 1.02 | 1.05 ± 1.02 | 0.95 ± 0.88 |
|  |  |  |  |
| Males 0-6 | 0.79 ± 0.91 | 0.69 ± 0.46 | 0.50 ± 0.36 |
| Males 7-12 | 0.81 ± 0.57 | 1.00 ± 1.52 | 0.74 ± 0.82 |
| Males 13-19 | 1.32 ± 1.03 | 1.08 ± 1.10 | 0.95 ± 0.95 |
|  |  |  |  |
| Females 0-6 | 1.17 ± 1.62 | 0.65 ± 0.31 | 2.32 ± 2.37 |
| Females 7-12 | 0.97 ± 1.55 | 0.62 ± 0.60 | 0.69 ± 0.74 |
| Females 13-19 | 1.13 ± 0.98 | 0.95 ± 0.74 | 0.96 ± 0.70 |
|  | | | |
| **Aripiprazole** |  |  |  |
| 0-6 | 3.07 (one case) | 2.26 ± 1.18 | 1.26 ± 0.92 |
| 7-12 | 8.12 ± 3.86 | 3.41 ± 2.80 | 2.49 ± 1.60 |
| 13-19 | 10.97 ± 6.01 | 5.94 ± 5.08 | 3.94 ± 3.28 |
|  |  |  |  |
| Males 0-6 | 3.07 (one case) | 2.22 ± 1.29 | 1.26 ± 0.92 |
| Males 7-12 | 8.32 ± 4.12 | 3.33 ± 2.42 | 2.51 ± 1.50 |
| Males 13-19 | 10.83 ± 5.93 | 6.69 ± 5.96 | 4.13 ± 3.33 |
|  |  |  |  |
| Females 0-6 | No cases | 2.5 (one case) | No cases |
| Females 7-12 | 7.25 ± 2.78 | 3.65 ± 3.69 | 2.38 ± 2.02 |
| Females 13-19 | 11.30 ± 6.37 | 4.96 ± 3.41 | 3.61 ± 3.17 |
|  | | | |
| **Quetiapine** |  |  |  |
| 0-6 | 253.93 (one case) | 46.71 ± 30.42 | No cases |
| 7-12 | 75.09 ± 91.49 | 31.55 ± 6.29 | 49.43 ± 42.59 |
| 13-19 | 91.55 ± 103.36 | 64.28 ± 76.97 | 42.60 ± 48.32 |
|  |  |  |  |
| Males 0-6 | No cases | 25.2 (one case) | No cases |
| Males 7-12 | 101.07 ± 117.10 | 31.55 ± 6.29 | 63.11 ± 44.19 |
| Males 13-19 | 96.16 ± 113.07 | 66.77 ± 88.76 | 50.74 ± 66.71 |
|  |  |  |  |
| Females 0-6 | 253.93 (one case) | 68.22 (one case) | No cases |
| Females 7-12 | 42.63 ± 38.88 | No cases | 28.90 ± 35.38 |
| Females 13-19 | 87.64 ± 95.46 | 62.63 ± 68.49 | 38.23 ± 34.11 |
|  | | | |
| **Olanzapine** |  |  |  |
| 0-6 | 4.58 ± 0.59 | 4.69 (one case) | No cases |
| 7-12 | 4.39 ± 0.82 | 2.94 (one case) | 5.68 ± 3.95 |
| 13-19 | 5.54 ± 3.62 | 5.65 ± 4.12 | 5.90 ± 4.30 |
|  |  |  |  |
| Males 0-6 | 4.59 ± 0.59 | 4.69 (one case) | No cases |
| Males 7-12 | 4.50 (one case) | No cases | 6.41 ± 4.16 |
| Males 13-19 | 6.13 ± 3.88 | 7.26 ± 5.31 | 7.25 ± 5.51 |
|  |  |  |  |
| Females 0-6 | No cases | No cases | No cases |
| Females 7-12 | 4.35 ± 1.00 | 2.94 (one case) | 2.75 (one case) |
| Females 13-19 | 4.71 ± 3.16 | 4.52 ± 2.59 | 4.94 ± 2.86 |
